# Carrier envelope phase sensitivity of photoelectron circular dichroism

**DOI:** 10.1039/d2cp03077b

**Published:** 2023-01-13

**Authors:** Václav Hanus, Sarayoo Kangaparambil, Martin Richter, Lukas Haßfurth, Martin Dorner-Kirchner, Gerhard G. Paulus, Xinhua Xie, Andrius Baltuška, Stefanie Gräfe, Markus Zeiler

**Affiliations:** a Photonics Institute, Technische Universität Wien 1040 Vienna Austria hanus.vaclav@wigner.hu markus.zeiler@tuwien.ac.at; b Wigner Research Centre for Physics, Institute for Solid State Physics and Optics 1121 Budapest Hungary; c Institute of Physical Chemistry and Abbe Center of Photonics, Friedrich Schiller University Jena, Helmholtzweg 4 07743 Jena Germany s.graefe@uni-jena.de; d Fraunhofer Institute for Applied Optics and Precision Engineering 07745 Jena Germany; e Institute for Optics and Quantum Electronics, Friedrich-Schiller-Universität Jena 07743 Jena Germany; f SwissFEL, Paul Scherrer Institute 5232 Villigen PSI Switzerland

## Abstract

We report on a combined experimental and numerical study of photoelectron circular dichroism (PECD) induced by intense few-cycle laser pulses, using methyloxirane as the molecular example. Our experiments reveal a remarkably pronounced sensitivity of the PECD strength of double-ionization on the carrier-envelope phase (CEP) of the laser pulses. By comparison to the simulations, which reproduce the measured CEP-dependence for specific orientations of the molecules in the lab frame, we attribute the origin of the observed CEP-dependence of PECD to the CEP-induced modulation of ionization from different areas of the wave functions of three dominant orbitals.

## Abstract 2

The PECD of methyloxirane molecules fragmenting along the laser propagation direction (left) shows a strong modulation by the CEP (top) due to the CEP-dependence of the ionization probability from different areas of specific orbitals (bottom right).

## Graphical abstract

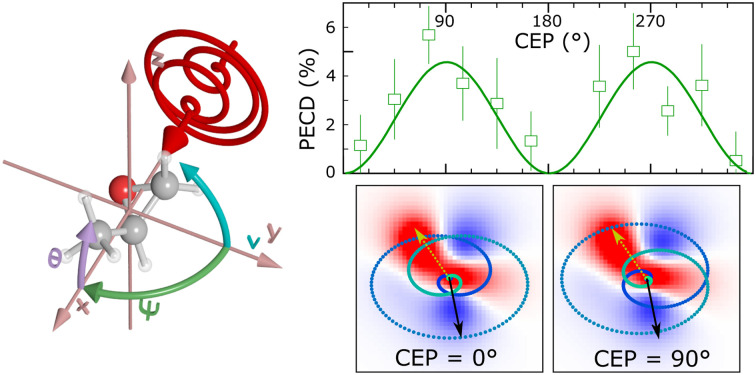

## 1 Introduction

The interaction of circularly polarized light and chiral molecules leads to an asymmetry of the photoelectron angular distribution (PAD) along the light propagation direction due to a phenomenon known as photoelectron circular dichroism (PECD).^1,2^ This forward–backward asymmetry due to PECD was detected for a wide range of interaction regimes ranging from single-photon ionization with extreme-ultraviolet (XUV) light,^3^ over multiphoton ionization^4^ to strong-field ionization^5^ with intense laser pulses. The origin and strength of the PECD effect can be explained by the scattering of the emitted electron on the effective chiral molecular potential.^6–10^

Strong-field ionization with ultrashort intense laser pulses is a particularly advantageous ionization regime since there the electron emission timing is linked to the laser field oscillations on an attosecond time scale, which opens up the possibility to control and image molecular processes using, *e.g.*, the laser intensity, pulse shape or carrier-envelope phase (CEP) as parameters.^11–13^ An important property of PECD is that the asymmetry in the PAD does not average to zero even when the ionizing lightwave interacts with randomly oriented samples of molecules. This renders PECD an effective tool to determine the enantiomeric excess of mixed molecular samples,^14^ or opens up the possibility to measure the chirality of an intermediate product of a chemical reaction on the femtosecond timescale.^15^

Traditionally, PECD is observed using light with two distinct helicities, *i.e.*, left or right circularly/elliptically polarized light. An interesting interaction regime in PECD is reached by the use of strong laser fields for which the helicity of the light varies within an optical cycle, such as cycle-sculpted two-color laser fields.^16,17^ Recent work investigated whether and to what extent PECD is affected by this sub-cycle variation.^18–20^

In contrast, the sensitivity of PECD to the sub-cycle variations of electron emission introduced by few-cycle pulses with a specific CEP^13,21,22^ has thus far been neglected. The key difference to two-color fields is that the CEP preserves the light's helicity throughout the whole pulse and does not provide instantaneous chirality,^20^ but merely modulates the electron emission timing.^23^ On the other hand, PECD in strong laser fields arises only during the early stages of the electron emission process.^18^ Whether the CEP has any influence on PECD, and if yes to what degree, is thus a highly interesting open question and constitutes the motivation for the present work.

In the strong-field regime, both the electron emission process and all subsequent electron acceleration mechanisms are non-resonant. Thus, it is in general not feasible to establish a direct correspondence between the ionizing molecular orbitals and certain bands in the photoelectron energy distributions. Instead, the molecular electronic structure is imprinted in the photoelectron momentum distributions.^24–26^ This is in contrast to single-photon^27^ and multiphoton ionization scenarios^15,28^ where the contribution from specific molecular orbitals can be distinguished based on photoelectron energy.

Therefore, in order to investigate the influence of certain laser pulse parameters on the emergence of the PECD in PADs in the strong-field regime, as is done here, specific experimental techniques need to be applied. One of the most sophisticated techniques suitable for this purpose is the detection of electrons and ions in coincidence. This allows obtaining photoelectron momentum distributions for a specific set of molecular fragment ions or charge states,^28–31^ a capability often referred to as coincidence imaging.

In this work, using coincidence imaging and the methyloxirane (propylene oxide) molecule, CH_3_CHCH_2_O, as the example, we show for the first time, to our knowledge, a dependence of the PECD-strength on the CEP of intense few-cycle laser pulses used for strong-field ionization. We show that both electrons emitted during a specific ionization-fragmentation reaction starting from the doubly charged molecular cation carry a CEP-dependent chirality. By extracting the molecular breakup axis from our coincidence data we show that the PECD is also sensitively dependent on the molecular orientation relative to the laser field's polarization plane. By comparison of the measured orientation-dependence of the PECD-strength to that obtained through numerical simulations, we attribute the origin of the observed CEP-dependence of PECD to the CEP-induced modulation of ionization from different areas of the wave functions of three dominant orbitals.

## 2 Experiments

Using a reaction microscope,^32^ also known as COLTRIMS, we measured in coincidence the three-dimensional momentum vectors of charged ions and electrons emerging from the interaction of methyloxirane molecules with intense few-cycle elliptically polarized pulses. One of the first investigations of PECD was performed using methyloxirane as the molecular sample.^27^ Subsequent works^30,31,33^ established this species as a benchmark molecule, since methyloxirane is one of the smallest chiral molecules and therefore amenable also to multi-electron simulations.

**Fig. 1:**
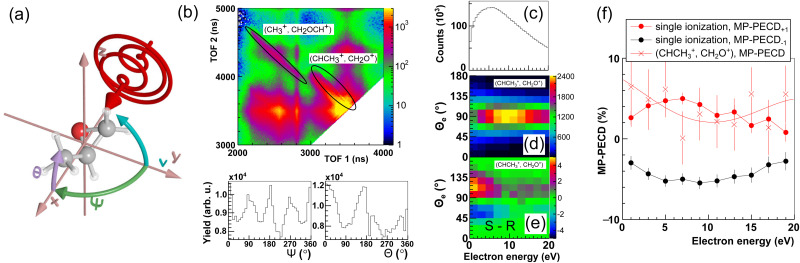
(a) Sketch of the geometry of the experiment defined by laser propagation direction *x*, propagation direction of the molecular jet *y* and spectrometer axis *z*. The molecular orientation in the such defined lab frame is defined by the Euler angles *Ψ* and *θ* of a specific ionic fragment. The third Euler angle *ν* (rotation of the molecule about its axis) is inaccessible in the experiment. (b) Top: Measured photoion–photoion coincidence plot. Time-of-flight in the spectrometer of the reaction microscope of one molecular fragment (TOF 1) *via* that of the second (TOF 2). Two-body ionization fragmentation reactions appear as parabolic lines. The two reactions discussed in the text are marked by black ellipses. In this work we focus on the one to the right, detailed in eqn (2). Bottom: Yield of the reaction eqn (2) over the Euler angles *Ψ* and *θ* defined in (a). (c) Photoelectron energy distribution for single ionization of methyloxirane. (d) Photoelectron energy distribution for the ionization-fragmentation reaction eqn (2) as a function of the electron emission angle *θ*_e_ in the lab frame. This angle is defined identical to the molecular orientation angle *θ* shown in (b). (e) MP-PECD_−1_ parameter in % calculated using eqn (4) as a function of the electron emission angle *θ*_e_ in the lab frame and photoelectron energy. (f) Dots show the MP-PECD parameter calculated using eqn (4) over photoelectron energy for single ionization for helicities +1 (red) and −1 (black). Crosses show the MP-PECD parameter calculated using eqn (5) for the ionization-fragmentation channel eqn (2). All data in the figure are integrated over the CEP and the molecular orientation (except for the data in panel (b) bottom, which are only integrated over CEP).

The laser pulses used for the experiments exhibited a broad spectrum centered around 750 nm and a full-width at half maximum (FWHM) duration in intensity of 4.5 fs. Pulses impinged on the target molecules with a repetition rate of 5 kHz. The propagation direction of the laser pulses was along the *x*-direction of the lab coordinate system, see the sketch in Fig. 1(a). The laser vector potential, from which the laser electric field is derived by 
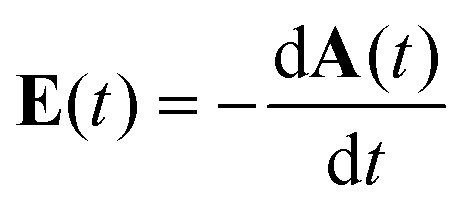
, in the polarization plane *y*–*z* at the point of interaction with the molecules had the form1
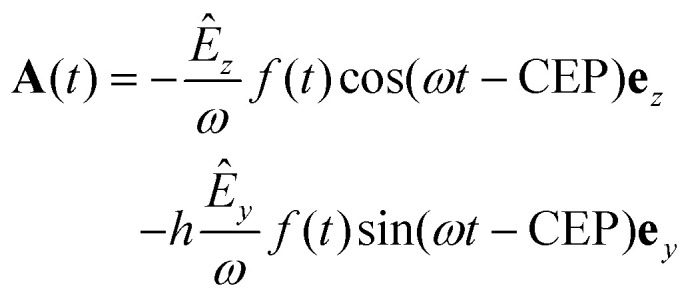
where *f*(*t*) denotes the pulse envelope normalized to 1, *ω* is the angular frequency corresponding to the spectral center wavelength 750 nm, *Ê*_*z*_ and *Ê*_*y*_ are the peak field strengths along *z* and *y*, respectively, CEP is the carrier-envelope phase, and *h* = {+1, −1} is the helicity of the elliptically polarized pulses, where *h* = +1 corresponds to right- and *h* = −1 to left-elliptically-polarized. The two helicities were created by passing linearly polarized pulses through a broad-band quarter waveplate, for two different rotations of the waveplate. To become independent of systematic experimental errors, PECD is usually defined as a normalized difference between PADs recorded for the two helicities +1 and −1 and/or two molecular enantiomers, see eqn (5) below.

**Fig. 2:**
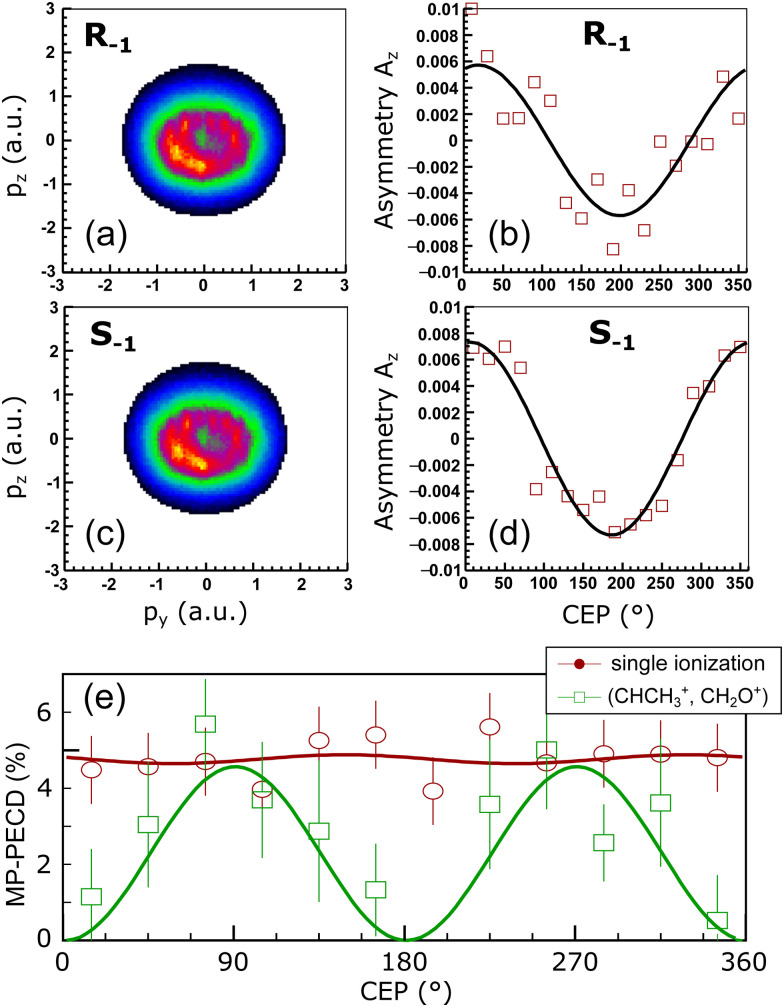
Measured data. (a) Momentum distribution in the laser polarization plane of photoelectrons emitted during single ionization of (*R*)-methyloxirane for helicity −1. Momentum is given in atomic units (a.u.). (b) CEP-dependence of the asymmetry *A*_*z*_, calculated using eqn (6), of the photoelectron momentum distribution in (a). Fit line is a guide to the eye. (c) Same as (a), but for (*S*)-methyloxirane. (d) Same as (b), for the momentum distribution in (c). (e) MP-PECD parameter eqn (5) over CEP for single ionization (open circles) and the ionization-fragmentation reaction eqn (2) (open squares) integrated over all molecular orientations. Fit lines are guides to the eye.

The pulse duration was monitored throughout the experiments by recording the so-called phase potato with a stereo above-threshold ionization phasemeter device.^34,35^ From the phase potato the CEP of the pulses was retrieved for each and every laser shot. With this method the CEP can only be obtained with respect to an unknown offset value. This offset value was calibrated such that the measured asymmetry of the momentum distribution of electrons emitted during single ionization of methyloxirane in the laser polarization plane featured a positive maximum for CEP = 0°, and a negative maximum for CEP = 180°, see Fig. 2(b) and (d). With this calibration, the measured CEP-dependence of the electron asymmetry agreed with that obtained from the simulations that will be described below.

The peak intensity of the pulses and their polarization states were characterized at the point of laser-molecule interaction by recording momentum distributions of singly ionized helium.^22^ By that we found that the peak intensity at the point of interaction was 5 × 10^14^ W cm^−2^ and the ellipticity *ε* = *Ê*_*y*_/*Ê*_*z*_ in the +1 and −1 helicities were 0.69 and 0.72, respectively.

The reaction microscope consists of a two-stage arrangement to provide an internally cold ultrasonic jet of methyloxirane molecules (propagating along −*y*-direction), which then drift into an ultra-high vacuum interaction chamber at a base pressure of 1 × 10^−10^ mbar. Electrons and ions were guided by weak magnetic (12.6 G) and electric fields (27 V cm^−1^) along the spectrometer axis (*z*-direction) to two position and time sensitive multi-hit detectors. The distances to the ion respectively electron detector from the point of interaction were 57 mm and 445 mm. Further details on the reaction microscope can be found in ref. 17, 36, 37 and on the optical setup in ref. 22.

Two enantiopure samples of methyloxirane, *S* and *R*, interacted with both light helicities, +1 and −1, resulting in four experimental combinations: *S*_+1_, *S*_−1_, *R*_+1_, *R*_−1_. To average over experimental random fluctuations and drifts of experimental conditions during the long measurement time (several days), the light helicities were automatically changed every 10 min, and every two hours the molecular enantiomeric samples were exchanged.

## 3 Extraction of PECD strength from measured data

Our measurements revealed that for the laser pulse parameters used in our experiments, single as well as double ionization of methyloxirane molecules takes place. Upon double ionization, a large fraction of the molecules dissociates or fragments. We identified two fragmentation channels where two singly charged fragments are created: (CH_3_^+^, CH_2_OCH^+^), where the methyl group is broken, and (CHCH_3_^+^, CH_2_O^+^) involving a breakup of the COC-ring structure that is responsible for the chirality of the molecule. The two channels are clearly visible and marked in the photoion–photoion coincidence plot in Fig. 1(b). Furthermore, we identified three dissociation channels involving the ejection of neutral hydrogen. These we did not analyze further since neutral particles are not detected by our detectors which impedes unequivocal coincidence-selection based on momentum conservation. In this work we focus on the reaction2
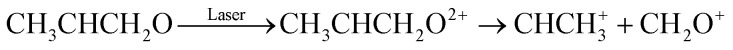
as it provided the best statistics out of all observed double ionization-fragmentation channels.

A decisive parameter in the investigation of laser-induced molecular fragmentation is the orientation of the molecule in the lab-frame, in which the laser field is defined. The orientation of the molecule at the time of the ionization is accessible in our experiment *via* the momenta of the two fragments. We reconstructed the orientation of the molecule assuming that the fragments created in the reaction eqn (2) are ejected (i) promptly upon ionization such that the molecule does not rotate significantly between laser-interaction and fragmentation (axial recoil approximation),^38^ and (ii) along the molecular axis that connects the centers of mass of the two fragments.

We characterize the such obtained orientation of the molecular axis in the lab frame by two Euler angles: the angle *θ* of the ejection direction of CHCH_3_^+^ with respect to the laser propagation direction *x*, and the angle *Ψ* of the ejection direction of CHCH_3_^+^ to the *y* direction, see Fig. 1(a) for a visualization. The third Euler angle *ν* that describes the rotation of the molecule about its axis is not accessible from our experimental data. However, as we will show using our simulations, the angle *ν* has a decisive influence on the measured PECD strength. Throughout the paper we will discuss the dependence of the PECD strength on the ejection angle of CHCH_3_^+^ within two specific planar cuts through the polar sphere: First, for variations of the angle *θ* in the *xz*-plane defined by *Ψ* = 90° and, secondly, for variations of the angle *Ψ* in the *xy* plane defined by *θ* = 0°. Adopting a geographical notation we denote the first plane by Meridian 90 and the second one by Equator.

To quantify the PECD effect, we calculated the so-called multi-photon PECD (MP-PECD) parameter^29^ from the forward–backward asymmetry of the momentum distribution of the first electron that is registered on the detector for a given laser shot. Note, that for double ionization this is not necessarily the electron that is first ejected from the molecule during the laser-ionization process. This forward–backward asymmetry emerges if either the photoelectron momentum distributions of the two enantiomers *S* and *R* measured for a specific helicity are compared, or when the photoelectron momentum distributions of a given enantiomer measured for the two helicities +1 and −1 are compared. The asymmetry can be quantified by the parameter3
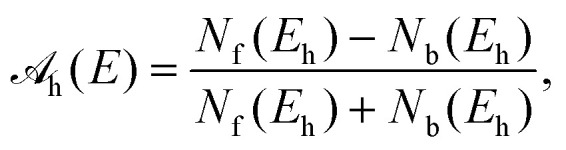
where *N*_f,b_(*E*_h_) is the number of detected electrons in the forward (f) respectively backward (b) direction for enantiomer *E* = (*S*,*R*) when helicity h (either +1 or −1) is used. Forward/backward is defined by the electron's momentum *p*_*x*_ along the laser pulse propagation direction *x*. The MP-PECD parameter for a specific helicity h is then derived from 𝒜_h_(*E*) as

4 MP-PECD_h_ = 2[𝒜_h_(*S*) − 𝒜_h_(*R*)].

To obtain better statistics, one can also use a parameter that combines the two measurements for the two helicities by averaging them:5
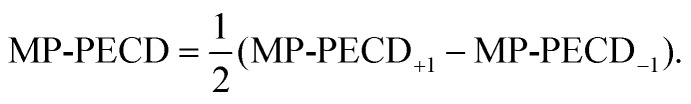


To eliminate possible artificial asymmetries in the electron momentum distributions, a careful post-processing was performed. Potential artificial PECD could, for example, arise from shortcomings of the experiment such as an unequal sensitivity of the multi-channel plates, or cross-talk of momentum components due to the effects of the magnetic field used in our apparatus. Also transformations of the momentum distributions performed during the data analysis, such as shifts along the *p*_*x*_ direction or rotations about the zero-momentum point, could result in artificial PECD. In the following we will illustrate the correctness of our data acquisition and processing.

We start with the PECD measured for single ionization. The photoelectron energy distribution for single ionization is shown in Fig. 1(c). It reaches its maximum around an electron energy of 7 eV. The MP-PECD_+1_ and MP-PECD_−1_ parameters for single ionization as a function of photoelectron energy, calculated using eqn (4), are depicted in Fig. 1(f) as dots. The data are mirror-symmetrical about zero within experimental error, as it should be.^1,2,33^

We now turn to the ionization-fragmentation reaction eqn (2). The energy distribution of photoelectrons emitted during the double ionization step is depicted in Fig. 1(d) as a function of the electron emission angle *θ*_e_. This angle in the lab frame is defined identical to the molecular orientation angle *θ*, see the sketch in Fig. 1(a). The photoelectron energy distribution of the fragmentation channel peaks at an energy of about 11 eV, slightly higher than the distribution for single ionization, and is centered around *θ*_e_ = 90°. Thus, the electron emission rate peaks parallel to the laser polarization plane.

To demonstrate the correctness of the measured PECD values for this channel, we plot in Fig. 1(e) the parameter MP-PECD_−1_, calculated using eqn (4), as a function of *θ*_e_ and photoelectron energy. The anti-symmetry of this distribution about the *θ*_e_ = 90° axis demonstrates the correctness of the PECD values measured for the ionization-fragmentation channel eqn (2).^9,39^ Moreover, the measured anti-symmetry also demonstrates that both electrons that become ejected during double ionization carry PECD. This is because in our analysis we exclusively consider the first electron that hits the electron detector, as mentioned above. However, this could be either the first or second electron that becomes ejected during the laser-ionization process. Although it is possible for certain values of the CEP to extract from the measured electron momentum distributions the information, which electron has been ejected first and which one second, as we could recently show,^13^ this assignment cannot be made in the present case for all values of the CEP. The distribution of the CEP-integrated MP-PECD_−1_ parameter in Fig. 1(e) is thus composed of a statistical mixture of first and second ejected electrons, and its asymmetry about *θ*_e_ = 90° proves that both electrons carry PECD, in agreement with earlier work, see *e.g.* ref. 33.

The total number of photoelectrons in the distribution in Fig. 1(e) consists of less than one tenth of electrons in the distribution for single ionization in Fig. 1(c), which reflects the small probability of the reaction eqn (2) to take place. To increase the statistical significance of the PECD values extracted from the double ionization momentum distributions, we will for the remainder of the paper use the MP-PECD parameter defined in eqn (5) that is averaged over both enantiomers and helicities. This parameter is plotted by crosses in Fig. 1(f) in comparison with the MP-PECD_+1_ and MP-PECD_−1_ values for single ionization.

## 4 CEP-dependence of PECD

We now turn to investigating the CEP-dependence of PECD. We start by discussing the up-down asymmetry of the photoelectron momentum distributions correlated with singly ionized (*R*)- and (*S*)-methyloxirane. These momentum distributions in the laser polarization plane are shown for helicity -1 of the laser field in Fig. 2(a) respectively Fig. 2(c), where we have integrated over all values of the CEP. The ellipticity of the laser field manifests itself in the donut shape of the electron momentum distributions.^21–23,40^ The normalized up-down asymmetries of these electron momentum distributions along the *p*_*z*_-direction,6
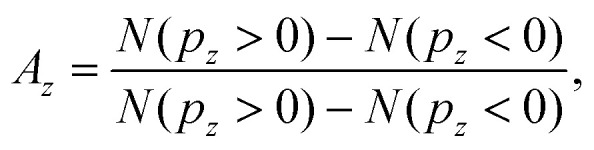
as a function of CEP are shown in Fig. 2(b) and (d). Here, *N*(*p*_*z*_ > 0) and *N*(*p*_*z*_ < 0) are the number of electrons with momentum *p*_*z*_ larger respectively smaller than zero. These asymmetries show, within experimental resolution, the same CEP-dependence for (*R*)- and (*S*)-methyloxirane. Hence, the different chirality of the two enantiomers does not lead to a different CEP-dependence of the electron emission asymmetry within the polarization plane. Instead, PECD manifests itself as a forward–backward asymmetry in electron emission perpendicular to the polarization plane, *i.e.*, along the laser propagation direction *x*.

If we plot the CEP-dependence of the forward–backward electron emission asymmetry along *p*_*x*_ for the ionization-fragmentation reaction eqn (2), defined by the MP-PECD parameter from eqn (5), we find a pronounced CEP-modulation of this quantity, see Fig. 2(e). The MP-PECD modulation features two maxima of about 5% at CEP values 90° and 270°, orders of magnitudes stronger than the one measured in photoabsorption circular dichroism experiments with weak narrow-band light. To our knowledge, this is the first demonstration of the CEP-dependence of PECD. Moreover, the measured CEP-modulation is surprisingly strong and reaches a modulation depth of almost 100%. In contrast, the MP-PECD parameter for single ionization of methyloxirane, also shown in Fig. 2(e), does not show any CEP-dependence within the experimental precision. An explanation for this observation will be provided below.

## 5 Influence of molecular orientation on PECD

Using the coordinate system defined above and depicted in Fig. 1(a), we will in the following explore the dependence of the PECD strength on the orientation of the molecule in the lab-frame, in which the laser field is defined. The molecular orientation in the lab-frame is in general crucial for the outcome of laser-induced molecular fragmentation reactions,^41^ but it is particularly important when the influence of the CEP on molecular fragmentation is of interest^23^ since the CEP is a quantity that is defined in the lab-frame. Here, as we are interested in the influence of the CEP on the PECD, the role of the molecular orientation is additionally important since (i) the molecule possesses a permanent dipole moment. In strong-field ionization, the ionization rate depends sensitively on the relative orientation of the molecular dipole vector and the (instantaneous) laser polarization vector. The latter is sensitive to the CEP. (ii) Different molecular orbitals (MOs) are ionized with different probability, both for energetic but also directional (orientational) reasons. (iii) The breakup channel of interest here is dominated by a few selected MOs, as will be shown below.

**Fig. 3:**
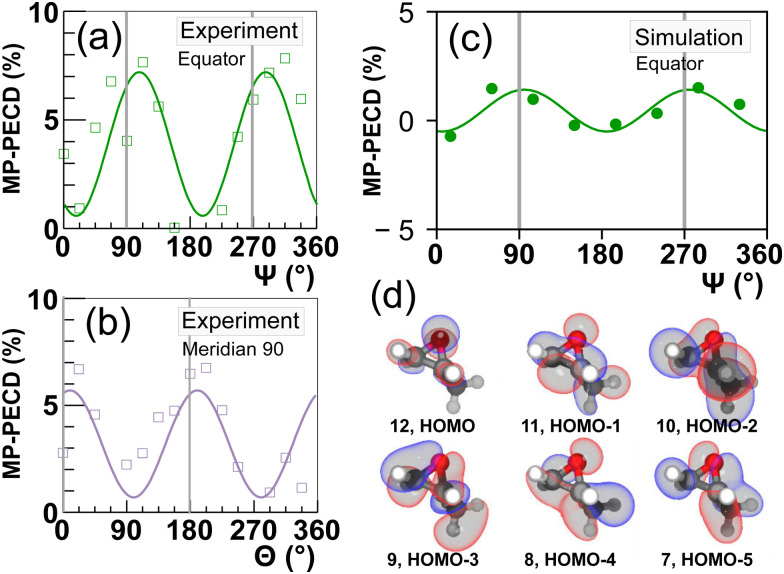
(a and b) The squares depict the measured MP-PECD parameter eqn (5) for the ionization-fragmentation reaction eqn (2) over the angle *Ψ* (a), *θ* (b). The other angle is restricted to a range −22.5° to +22.5° in both cases, and the angle *ν* is integrated over. (c) MP-PECD parameter eqn (5) obtained from the simulations, as described in the text, over *Ψ*, with |*θ*| ≤ 35°, integrated over the angle *ν*. The data are integrated over the CEP in all figures. The lines represent sine squared fits to the measured data and are only meant to guide the eye. The vertical gray lines in (a–c) mark the angles where the molecule fragments along/against the laser propagation direction (*Ψ* = 90°/270° and *θ* = 0°/180°). Small deviations of the peaks of the data fit lines from the position of the gray lines are merely a result of the fit and have no statistical relevance. (d) Visualization of the 6 most loosely bound orbitals of methyloxirane.

Fig. 3(a) and (b) show the MP-PECD value for the measured data obtained using eqn (5) as a function of the CHCH_3_^+^ ejection angle in the Equator and Meridian 90 planes, respectively. To ensure that the fragmentations happen in these planes, the CHCH_3_^+^ ejection angle orthogonal to it was restricted to ±22.5°. It can be seen that the MP-PECD value is almost zero when the breakup axis lies in the laser polarization plane *yz*. In contrast, when the breakup axis is directed along the laser propagation direction, marked by gray vertical lines in Fig. 3, the MP-PECD value reaches more than 7% and is even higher than the MP-PECD value of 5% of the single ionization case shown in Fig. 1(f). By comparison of these findings to the shapes of the most loosely bound molecular orbitals of (*R*)-methyloxirane, depicted in Fig. 3(d), this orientation dependence suggests the conjecture that the strength of the PECD originates from the involvement of different orbitals in the breakup.

To support this conjecture, we performed a large set (200 in total) of real-time real-space time-dependent density functional theory (rt-TDDFT) calculations to simulate the interaction of (*R*)-methyloxirane with a few-cycle pulse with parameters as in the experiment for the two helicities +1 and −1, involving 50 orientations for four different values of the CEP, respectively. In every simulation run, the orientation of the molecule and CEP was randomly defined. For the simulations, we have employed the OCTOPUS code.^42,43^ The total amount of core hours for these calculations was about 640 000. We explicitly included the interaction with the 12 highest occupied molecular orbitals, *i.e.* 24 electrons, and calculated the resulting photoelectron momentum distributions and the orbital depopulation probability at the end of the laser interaction.

From the forward–backward asymmetry of the photoelectron distributions the MP-PECD was calculated using eqn (5). By selecting from all simulated orientations those runs where |*θ*| ≤ 35° and averaging over all values of the CEP, we calculated the MP-PECD value in the Equator plane as a function of the angle *Ψ*. The result is depicted in Fig. 3(c) and shows that the simulations predict a similar *Ψ*-dependence of the MP-PECD strength as in the experiment: the maximum value of MP-PECD is obtained when the molecule is aligned along the laser propagation direction *x*.

## 6 Orbital contributions

**Fig. 4:**
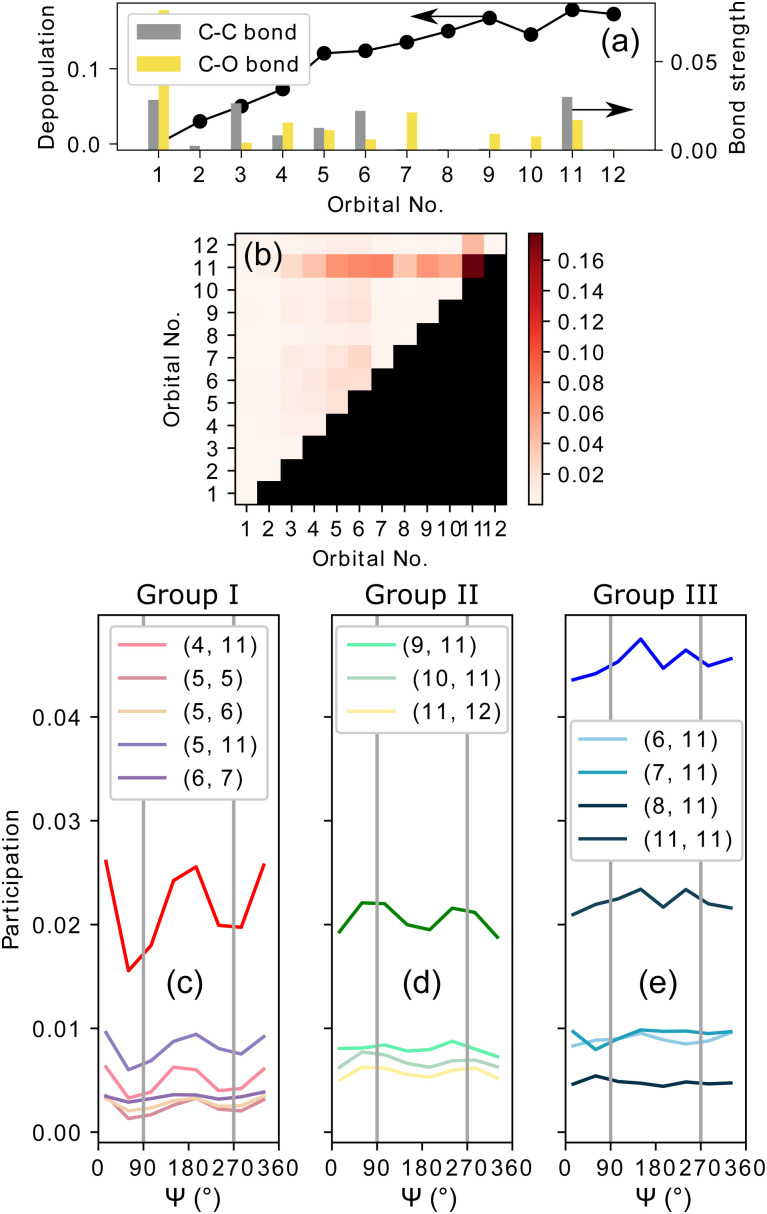
Results of simulations. (a) Full circles represent the depopulation of orbitals after the laser-molecule interaction averaged over all considered CEPs. Gray and yellow bars show the bond strength of an orbital in the two bonds C–C and C–O, respectively. These bonds need to be broken in the ionization-fragmentation reaction eqn (2). (b) Color encoded matrix of the product of bond-strength and orbital depopulation, referred to as breakup participation, for all combinations of orbitals considered in the simulations. See text for details. Values in (a and b) are averaged over all molecular orientations. (c–e) Breakup participation values from (b) for three groups, denoted as I, II and III, of selected orbital combinations (indicated in the figures) over *Ψ* with |*θ*| ≤ 35°. The vertical gray lines mark *Ψ* = 90°/270°, where the molecule fragments along/against the laser propagation direction.

Let us now scrutinize the origin of the angular dependence of the MP-PECD strength. For the breakup into two singly charged cations (CHCH_3_^+^, CH_2_O^+^) along ionization-fragmentation reaction eqn (2), two electrons need to be removed from the molecule and two specific bonds, a C–C and a C–O bond, need to be broken. We start our analysis by evaluating from which MOs electron density needs to be removed to break these two bonds. To this end, we depict in Fig. 4(a) by the gray and yellow bars the simulated contribution of each of the 12 considered orbitals to the C–C respectively the C–O bond. We denote this quantity by bond strength.

The data show that the molecule can break with high probability when, for example, two electrons are removed from orbital No. 11. Another possibility is to remove one electron from orbital No. 6 and the other one from No. 7. Yet another possibility is to remove two electrons from orbital Nr. 1. However, as is depicted by the black line in Fig. 4(a), orbitals with smaller index number that are more strongly bound, become accordingly less and less depopulated during the interaction with the laser pulse. Thus, a strong contribution to the breakup-channel eqn (2) necessitates that the two electrons are removed from orbitals that feature both a high C–C and C–O bond strength, and at the same time become strongly depopulated during the laser interaction.

Both requirements can be evaluated by calculating the product of the bond strength and the orbital depopulation for all (12 + 1) × 12/2 = 78 possible combinations of removing two electrons from 12 orbitals. This product, which we denote as breakup participation, is depicted in Fig. 4(b) in the form of a matrix, where the value of the product is encoded by false color. High values of this product indicate that the corresponding two orbitals contribute strongly to the C–C and C–O bonds and are strongly depopulated by the laser field, hence, are important for the considered breakup-channel eqn (2). By selecting orbital combinations that feature a large product value in the matrix, we can thus analyze their importance in the formation of the PECD. Since the MP-PECD is strongly dependent on the orientation of the molecule in the lab-frame, in particular on the angle *Ψ* in the equator-plane, as we have shown in Fig. 3, we will in the following analyze the angular-dependence of the ionization-fragmentation probability in this plane for the most important orbital-combinations that feature a high product value in Fig. 4(b).

Fig. 4(c)–(e) show the results of this analysis. In these figures we have sorted the orbital combinations with the highest product values into three groups, where each group is in total responsible for about a third of all ionization-fragmentation reactions. To ensure fragmentations in the equator-plane, only simulations where the molecular axis was aligned within |*θ*| ≤ 35° were considered for these figures. Group I, shown in Fig. 4(c), consists of orbital combinations that contribute to the fragmentations by the highest amount, when the breakup axis lies in the laser polarization plane (*Ψ* ≈ 0° or 180°).

Group II, shown in Fig. 4(d), consists of orbital combinations that feature the opposite angular dependence: They are involved in ionization-fragmentations that happen most strongly perpendicular to the laser polarization plane (*Ψ* ≈ 90° or 270°). As the measured PECD strength in Fig. 3(a) shows the same angular-dependence, we can conclude that at least some orbitals in Group II (Nr. 9 to 12) are carriers of the measured MP-PECD. Since the loosely bound orbital Nr. 11 is involved in all three groups I to III, we can conclude that this orbital is rather unspecific. Orbitals Nr. 9, 10 and 12, however, only contribute to the angular-dependence of group II. Therefore, we claim that these orbitals are carriers of the measured MP-PECD.

In contrast, the orbital combinations in group I (Nr. 4 to 7) cause an anti-phase angular dependence of the ionization-fragmentation probability as compared to those in group II. As this is opposite to the measured dependence, we can conclude that orbitals Nr. 4 to 7 are not carriers of the measured MP-PECD. This is further corroborated by the fact that some of them also appear in group III, into which we have sorted all orbital combinations, which lead to an angular dependence that is either flat or does not coincide with the measured MP-PECD dependence, see Fig. 4(e).

## 7 Discussion of CEP-dependence of PECD

**Fig. 5:**
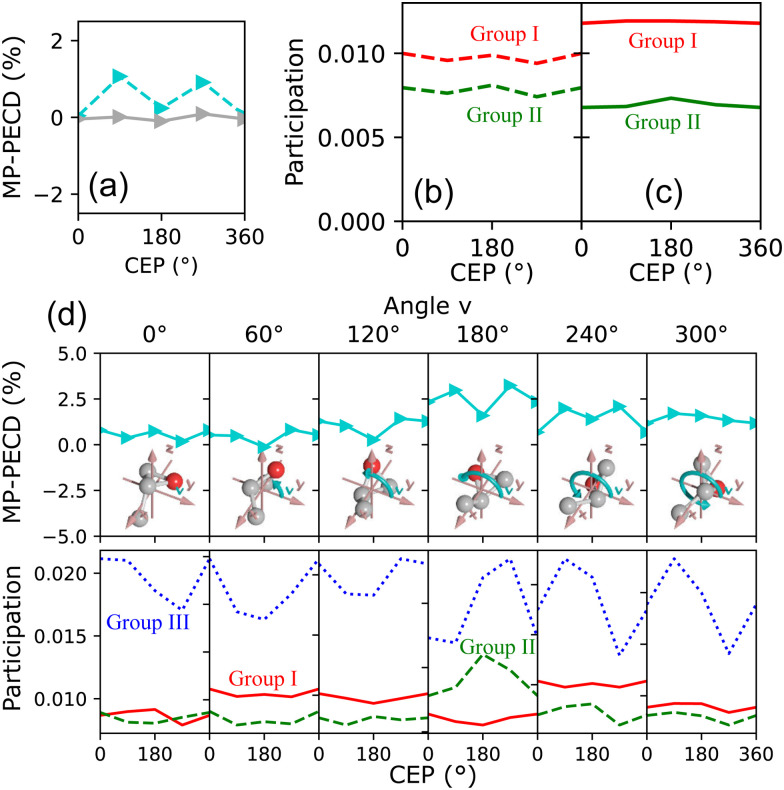
Results of simulations. (a) MP-PECD parameter from eqn (5) as a function of CEP. The dashed cyan line is for molecules aligned within 17.5° around the laser propagation axis *x*. The full gray line depicts the same value integrated over all molecular orientations. The data are integrated over the angle *ν* in both cases. (b) Participation (as defined in the caption of Fig. 4) of orbital groups I and II corresponding to the dashed line in (a). (c) Participation as in (b), but for the full line in (a). (d) Upper row: MP-PECD parameter over CEP for certain values of the angle *ν* (indicated above the separate panels). Lower row: Participation of orbital groups I, II and III corresponding to the data in the upper row.

To understand the origin of the measured CEP-dependence of the MP-PECD parameter, we resort to the simulations. In Fig. 5(a) we plot the simulated dependence of the MP-PECD parameter over the CEP. Whereas the CEP-modulation nearly vanishes for randomly oriented molecules (full gray line), the MP-PECD shows a CEP-modulation comparable to the measured one (dashed cyan line) when only molecules oriented within 17.5° to the laser propagation direction *x* are selected, *i.e.*, around *Ψ* = 90°, *θ* = 0°. As the measured angular distribution of the ionization-fragmentation probability of reaction eqn (2) features pronounced maxima for molecules exactly for these angles (see Fig. 1(b) bottom), this selection mimics the situation of the experiment.

Fig. 5(b) and (c) show the CEP-dependence of the participation numbers, defined above in connection to Fig. 4, of orbital groups I and II corresponding to the data in Fig. 5(a). While the participation values do not feature a pronounced CEP-dependence, the relative strength of group II in comparison to group I is markedly higher when the molecules are oriented along the laser propagation direction, shown in Fig. 5(b), as compared to the case where the molecules are randomly oriented, shown in Fig. 5(c). As the former case corresponds to the situation of the experiment, these data indicate that the measured CEP-dependence of the MP-PECD parameter is connected to the relative contributions of the group II orbitals to the overall ionization-fragmentation yield.

This conclusion is in full agreement with the results obtained above, where we analyzed the orientation dependence of the orbital contributions to the overall ionization-fragmentation yield, see Fig. 4. There, we concluded by comparison to the measured MP-PECD orientation dependence, that the orbitals in group II are the carriers of the PECD. Thus, a relative enhancement of these orbitals leads to an increase of the MP-PECD values. Such enhancement takes place when the molecule is aligned perpendicular to the laser polarization plane, *i.e.*, along the laser propagation direction. This is exactly the orientation for which we measure the highest yield of the ionization-fragmentation reaction eqn (2), see the bottom panel of Fig. 1(b), even though the molecules are randomly oriented within the laser focus. Since the ionization-fragmentation probability is highest, when the molecules are aligned perpendicular to the laser polarization plane, in our experiment we dominantly select those molecules that are aligned perpendicularly to the laser polarization plane. Hence, in our experiment we measure a high value of the MP-PECD parameter even for initially randomly oriented molecules, *cf.*Fig. 1(f) and 2(e). Moreover, we surmise that also the single ionization probability maximizes for this orientation, which would explain the very similar values of MP-PECD for this channel as for the double-ionization channel. This is because either electron emitted from the group II orbitals carries PECD, as explained above.

Having established that in our experiment we detect mostly electrons from molecules oriented along the laser propagation *x*, for which the contributions of the group II orbitals are relatively enhanced, we focus in the following on this case. With that setting, the only free parameter of the experiment is the Euler angle *ν*. This angle marks the orientation of the methyloxirane molecule's oxygen atom within the laser polarization plane.

The simulated data in Fig. 5(d) visualizes the dependence of the MP-PECD strength on *ν*. The upper row depicts the MP-PECD over CEP for selected angles of *ν*, and the lower row shows the corresponding participation numbers of the group I, II and III orbitals. It can be clearly seen that both the average value of the MP-PECD parameter and its CEP-dependent modulation depth strongly depend on *ν*. For *ν* = 180° both quantities feature a maximum, whereas both quantities minimize for *ν* = 0°/360°. Moreover, the relative contributions of the group II orbitals in the bottom row panels show the same *ν*-behavior, confirming the above found conclusions on their role for the CEP-dependence of the MP-PECD.

If the simulated data in Fig. 5(d) are integrated over *ν*, the average MP-PECD value becomes smaller than for *ν* = 180° but the CEP-dependence with maxima at CEP = 90°/270° stays visible, see Fig. 5(a), and closely resembles the measured CEP-dependence shown in Fig. 2(e). As the angle *ν* is not accessible in our experiment, we cannot evaluate the increase of the CEP-averaged MP-PECD strength for specific values of *ν* that is predicted by the simulations. The reason is that in a coincidence fragmentation experiment, such as ours, at least three ionic fragments and correspondingly a triple-ionization process are necessary to fully establish the orientation of the molecule in the lab space. Therefore, the extraction of *ν* from the fragmentation reaction eqn (2) that starts from double ionization, is intrinsically impossible in such an experiment. Hence, a different approach, for example by extending two-color field-free molecular orientation^44^ to the three-dimensional case using two-dimensional waveforms,^16^ would need to be adopted.

Although the simulated data in Fig. 5(d) elucidate the important role of the orientation of the oxygen molecule, determined by the angle *ν*, they do not provide evidence for the origins of the CEP-modulation of the MP-PECD parameter that we observe both in our experiment and simulations. The participation of the decisive group II orbitals, shown in the lower row panels, does not reflect the CEP-modulation with maxima at CEP = 90°/270°—not even for the strongest case of *ν* = 180°. The participation numbers of the group II orbitals alone can, thus, not explain the CEP-dependence of the PECD.

### 7.1 Origins of the measured CEP-modulation

To understand the origins of the measured CEP-modulation of the PECD in Fig. 2(e), we consider the decisive role of the orientation of the methyloxirane molecule's oxygen atom within the laser polarization plane (determined by the angle *ν*) which becomes obvious from Fig. 5(d). Since the orientation of the oxygen atom signifies the orientation of the COC-ring structure that is responsible for the chirality of the molecule, the angle *ν* relative to the CEP determines which portions of the wave function are sampled by ionization. This is because the CEP defines the angles within the laser polarization plane *y*–*z* where the temporal maxima of the electric field strength, respectively those of the vector potential, can be observed.^22,23^

**Fig. 6:**
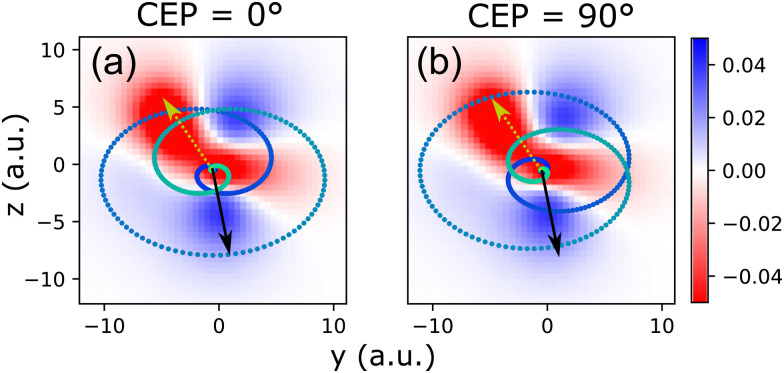
Trace of the normalized laser electric field vector (dotted line) within the polarization plane *y*–*z* and a false-color representation of a cut through the electronic wave function of the HOMO of methyloxirane in this plane (scaled in atomic units, a.u.) for the case *ν* = 0°, *Ψ* = 90°, *θ* = 0°. Time evolves from dark blue to light green in the trace of the laser field. The black and brown arrows indicate the laser electric field vector at two instants of time, at which they point towards two high-density regions of the wave function. The arrows are of identical length in (a) and (b). The black arrow shows that for CEP = 0° the electric field strength is stronger than for CEP = 90° when it samples the electron density in the blue area at *z* < 0. Thus, given the exponential sensitivity of the ionization probability to the electric field strength, this part of the wave function becomes ionized with higher probability for CEP = 0°. In contrast, the field strength is larger for CEP = 90° at the instant indicated by the brown arrow. Thus, the red area at *z* > 0 becomes ionized with higher probability for CEP = 90°.

The relation between the CEP-dependent angles under which the maxima of the laser field strength in the laser polarization plane are reached and the shape of the electronic wave function from which electrons are released is depicted in Fig. 6. For a given instant within the laser pulse, the probability of observing an emitted electron with momentum **k** is, within the strong-field approximation (SFA), determined by the ionization matrix element in combination with the action integral. The SFA ionization matrix element can be written as^45^

7

<svg xmlns="http://www.w3.org/2000/svg" version="1.0" width="27.454545pt" height="16.000000pt" viewBox="0 0 27.454545 16.000000" preserveAspectRatio="xMidYMid meet"><metadata>
Created by potrace 1.16, written by Peter Selinger 2001-2019
</metadata><g transform="translate(1.000000,15.000000) scale(0.015909,-0.015909)" fill="currentColor" stroke="none"><path d="M320 840 l0 -40 -40 0 -40 0 0 -160 0 -160 40 0 40 0 0 -40 0 -40 40 0 40 0 0 120 0 120 -40 0 -40 0 0 80 0 80 80 0 80 0 0 -40 0 -40 40 0 40 0 0 -240 0 -240 -40 0 -40 0 0 -40 0 -40 -80 0 -80 0 0 -80 0 -80 80 0 80 0 0 40 0 40 40 0 40 0 0 40 0 40 40 0 40 0 0 240 0 240 40 0 40 0 0 80 0 80 80 0 80 0 0 -320 0 -320 -80 0 -80 0 0 -80 0 -80 80 0 80 0 0 40 0 40 40 0 40 0 0 80 0 80 40 0 40 0 0 240 0 240 -40 0 -40 0 0 40 0 40 120 0 120 0 0 -400 0 -400 80 0 80 0 0 40 0 40 -40 0 -40 0 0 240 0 240 40 0 40 0 0 120 0 120 -40 0 -40 0 0 40 0 40 -320 0 -320 0 0 -40 0 -40 -40 0 -40 0 0 40 0 40 -120 0 -120 0 0 -40z"/></g></svg>

(**k**, *t*, *n*) = 〈**k** + **A**(*t*)|**r**·**E**(*t*)|*Ψ*_*n*_〉,

where 〈**k** + **A**(*t*)| is the final electronic state, which, according to the SFA, is a plane wave, **r**·**E**(*t*) is the dipole moment and |*Ψ*_*n*_〉 is the wave function of the *n*th electronic state from which ionization takes place. The matrix element and therewith the ionization probability |𝔐(**k**, *t*, *n*)|^2^ depends on the magnitude and angle of the vectorial electric field strength **E**(*t*) within the laser polarization plane relative to the shape of the electronic wave function. The latter, for a given molecular alignment *Ψ*, *θ*, is determined by the angle *ν*. Fig. 5 depicts two exemplary cases for a molecular orientation *Ψ* = 90° and *θ* = 0° [which is dominant in our experiment, *cf.*Fig. 1(b)] and the HOMO wave function (*n* = 0), interacting with pulses that exhibit CEP = 0°, respectively CEP = 90°. The angle *ν* is chosen as zero in both cases.

The key point that becomes obvious from Fig. 6 is that for different values of the CEP, directions where high electron density resides in the considered electronic state *n*, are reached at different times within the pulse and therewith at different field strength. Since strong-field ionization is exponentially sensitive to field strength, this results in strong CEP-modulation of the ionization process.^23^ As a result, the CEP becomes a crucial parameter that introduces sensitivity to the shape of the wave function, to its sign and, moreover, also to the electronic state *n* from which ionization takes place. When the laser electric field vector for a given CEP features large magnitudes under angles (equivalent with instants of time within the pulse), where the wave function of a specific state *n* features high electron density, the ionization matrix element |𝔐(**k**, *t*, *n*)|^2^ will be large. This way, ionization from states with *n* > 0 (HOMO−1, HOMO−2, *etc.*) can become dominant over ionization from HOMO.^11,46,47^ In the present case, ionization from lower-lying HOMO−*n* orbitals with *n* up to about 6 is comparably strong to that from the HOMO, as can be inferred from the orbital depopulation shown in Fig. 4(a) (black dots).

As a result, the relative orientation (determined by *ν*) of the molecular electronic wave function of a given state *n* to the peaks of the laser electric field in the polarization plane (determined by the CEP), dictate, *via* the dipole element 𝔐(**k**, *t*, *n*) in combination with the action integral phase-factor, the momentum of the emitted electrons, in particular their value along the laser propagation direction *z*, *k*_*z*_. As this value determines the observed PECD, the described connection between the CEP and the angle *ν* might be (one of) the reason(s) underlying the CEP-modulation of the PECD strength.

A possible scenario for the double ionization dynamics during reaction eqn (2) that is in agreement with all of the above is the following: During the rising edge of the laser pulse the first electron is released from one of the more loosely bound orbitals (small *n*) at an instant, when the laser electric field vector is aligned with an area of high electron density in this state, *cf.*Fig. 6. Importantly, to observe the fragmentation reaction eqn (2), the electron must be released from one of the orbitals that contribute strongly to the bonds that need to be broken in this reaction, *cf.* bond-strength in Fig. 4(a). Thus, the first ionization step selects from the randomly oriented molecular sample within the laser focus a suitably orientated molecule that can fulfill these constraints. Particularly important in this process is the angle *ν*, as this angle is intimately connected to the CEP-dependence of the PECD-strength, as we have shown above.

As time evolves within the pulse, the laser electric field increases and at an instant where it is suitably strong the second electron is released. Due to the ellipticity of the laser field this most probably happens a certain number of half-cycles after the first electron emission step.^13^ As a result, the laser field vectors at the two ionization instants form a pair with a fixed angle between them. Only if the laser electric field vector at the second instant is aligned with an area of large density in the wave function of a (more deeply bound) orbital that matches the first emptied orbital according to the matrix in Fig. 4(b), and, additionally, its magnitude is also large enough such that it leads to high ionization probability for this orbital, will the fragmentation reaction eqn (2) take place and a high value of the MP-PECD parameter will be measured.

As the CEP determines the relation (angle and magnitude) of the laser electric field vectors at both ionization instants, *cf.*Fig. 6, these many constraints are only fulfilled for certain values of the CEP. As a result, only for certain values of the CEP we measure in our experiment a large value of the MP-PECD parameter. For other CEP-values at least one of the constraints is not fulfilled by the laser electric field vectors at the two ionization instants and electron emission takes place from an orbital that leads to a small MP-PECD parameter. Even though this scenario can explain the measured CEP-modulation in Fig. 2(e), we need to leave a rigorous proof of this conjecture to future theoretical and experimental work.

We note that this scenario can also explain the CEP-insensitivity of the PECD parameter for single ionization that is visible in Fig. 2(e): It necessitates two ionization steps to select the rotation angle *ν* of the molecule about its own axis (out of a randomly oriented ensemble) and additionally to match it to the evolution of the laser electric field vector in the lab frame. The latter is determined by the CEP. Therefore, the two frames (the molecular rotation *ν* and the field evolution) cannot be matched by only one ionization step. As a result, in the single ionization case one effectively averages over all angles or, equivalently, all values of the CEP and, hence, the PECD parameter for single ionization becomes uncorrelated to the CEP.

## 8 Summary

In conclusion, we presented the first, to our knowledge, investigation of the CEP-dependence of PECD in the strong-field regime for a specific molecular ionization-fragmentation reaction, using the methyloxirane molecule as the example. Our experiments reveal a remarkably pronounced CEP-sensitivity of the PECD strength of this double-ionization reaction even when the molecules are randomly oriented in the lab frame. Employing reaction microscopy in our experiments, we furthermore succeeded in measuring the PECD strength as a function of the molecular orientation in the lab frame. This enabled us, by comparison of the measured data to the results of large-scale time-dependent density functional theory simulations, to trace the origin of the measured CEP-dependence of PECD to the dominant contributions of only three orbitals, and to reveal the crucial role of the orientation of the chirality-determining COC-ring structure within the laser polarization plane. Based on considerations guided by strong-field ionization theory we attribute the observed CEP-dependence of the PECD strength to the modulation of transition amplitudes between different areas of the wave function of the three dominantly contributing orbitals and the experimentally observed electron momentum **k**. This modulation is dictated by the orientation of the molecular orbitals relative to the laser electric field evolution in the lab frame, which is determined by the CEP.

## Conflicts of interest

There are no conflicts to declare.

## Supplementary Material
